# Analysing photonic structures in plants

**DOI:** 10.1098/rsif.2013.0394

**Published:** 2013-10-06

**Authors:** Silvia Vignolini, Edwige Moyroud, Beverley J. Glover, Ullrich Steiner

**Affiliations:** 1 Cavendish Laboratory, University of Cambridge, JJ Thomson Avenue, Cambridge CB3 0HE, UK; 2 Department of Physics and Astronomy, University College London, Gower Street, London WC1E 6BT, UK; 3 Department of Plant Sciences, University of Cambridge, Downing Street, Cambridge CB2 3EA, UK

**Keywords:** structural colour in plants, spectroscopy, iridescence, plant cuticle, multilayer interference

## Abstract

The outer layers of a range of plant tissues, including flower petals, leaves and fruits, exhibit an intriguing variation of microscopic structures. Some of these structures include ordered periodic multilayers and diffraction gratings that give rise to interesting optical appearances. The colour arising from such structures is generally brighter than pigment-based colour. Here, we describe the main types of photonic structures found in plants and discuss the experimental approaches that can be used to analyse them. These experimental approaches allow identification of the physical mechanisms producing structural colours with a high degree of confidence.

## 1. Introduction

Structural colour in nature is typically associated with the animal kingdom [1]. Diverse typologies of photonic structures including ordered [2,3], quasi-ordered [4] and completely random morphologies [5] have been reported in a range of animal species, such as butterflies, beetles, jellyfishes and birds. Examples of photonic structures in plants are much more rare and many of them have been described only very recently. These include a variety of flowers [6–9], leaves [10] and fruits [11,12]. The biological functions of these photonic structures are only starting to be unveiled: the ability of a flower to produce strong and intense coloration may facilitate pollination, as bumblebees can use iridescence as a cue to detect flowers  [6,8]. The functional significance of structural colour in leaves is, on the other hand, not fully understood [10,13,14]. Waxy and hairy structures protect the photosynthetic elements from ultraviolet (UV) radiation [15,16]. In fruits, a particular bright coloration could serve as an advertisement to attract seed dispersers, capitalizing, for instance, on the attraction of some birds to shiny objects [11,17]. The shape and the anatomy of plant surface topography not only affect their visual appearance and control the amount of light coupled into lower lying tissue, they also influence a range of other epidermal properties in a multi-functional fashion [18]. As an example, the conical epidermal cells found on the petals of most flowering plants enhance their coloration, temperature, pollinator grip and enhance wettability [19,20]. Similarly, a thick and waxy cuticle not only protects leaves from damage caused by UV radiation but also regulates water evaporation from these tissues [15,16]. In this review, we focus on the optical response of plant tissue. In particular, we review the main mechanisms of structural colour in flowers, leaves and fruits, and the most common optical techniques used to characterize these optical phenomena.

## 2. Investigation of structural colour in plants

Biological structures are anatomically and compositionally more complex than most fabricated optical elements. A complete characterization of the optical response of biological photonic systems, therefore, generally requires the combination of several optical methods. Plant tissues typically consist of more than one cell type, each with their own morphology and biochemical constituents. Plant surfaces are therefore highly heterogeneous and the description of the optical response needs to take into account a complex distribution of refractive indices and absorption. Moreover, the shape and dimensions of epidermal cells (surface layer) strongly influence the way that light is scattered and consequently impact on the visual appearance of the tissue.

### 2.1. Macro- and microscatterometry techniques

**Figure 1.**
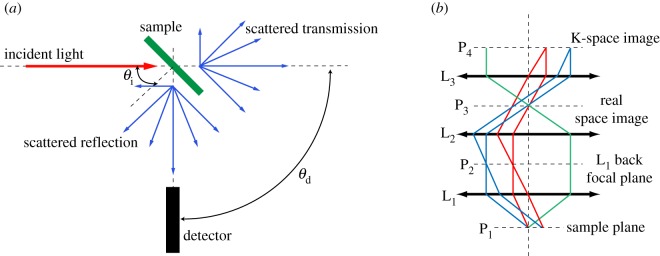
Macro- and microscatterometry techniques. (*a*) Goniometre set-up. A collimated beam of light macroscopically illuminates the sample at an angle of incidence *θ*_i_ that can be varied from 0° (normal incidence) to about 90°. The scattered light in transmission and reflection is collected for different angles *θ*_d_. (*b*) Conoscopic imaging principle. Light from the sample (illuminated in reflection or transmission) is collected by lens L_1_ with high NA. With the sample in the focal plane P_1_ of L_1_, the light rays (represented in different colours) are focused on the back focal plane P_2_. The second lens L_2_ is mounted in telescopic configuration with L_1_ to create an image of the sample in plane P_3_. The lens L_3_ is again mounted in telescopic configuration with L_2_ to form an image of the back focal plane of L_1_, P_2_ in P_4_. For simplicity, all lenses are drawn with the same focal length, and the distances between lenses correspond to the double of their focal length.

The optical response averaged over large surface areas is generally measured using an optical goniometer (figure 1*a*). In this method, a collimated beam from a light source illuminates the sample at an angle *θ*_i_ (which is varied by rotating the sample) and the scattered light in reflection and transmission is collected at *θ*_d_ (which is varied by rotating a detector arm). The illumination beam is generally provided by a broad-band light source. In the case of flowers, for example, the illumination spectral range should match the spectral response of the photoreceptors of the pollinators that visit this flower. This typically includes the UV and part of the visible (VIS) spectrum. Particular care has to be taken not to damage the plant tissue: high illumination intensities or prolongated exposition can, in fact, alter the tissue. Lamps with stable intensities and flat wavelength profiles are therefore preferred. Xenon and deuterium lamps are typically used to investigate the spectral properties in the UV region, while a combination of deuterium and tungsten lamps is used for UV to near-infrared spectroscopy [9]. A set of lenses and pinholes on the detector arm regulate the detected intensity and the angular resolution (typically ≈1°) and couple the light into an optical fibre. The core of the fibre also influences the detected angular resolution and intensity. The light from the fibre is coupled into a monochromator followed by a detector.

**Figure 2.**
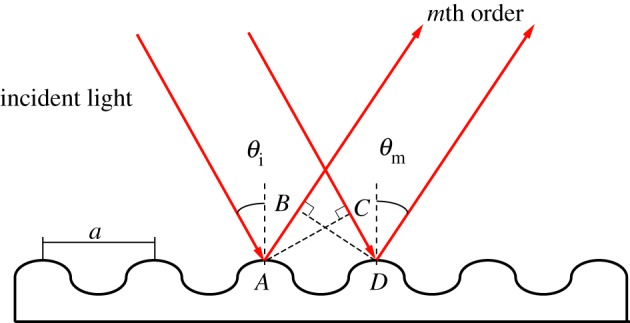
Diffraction gratings. The distance 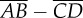 has to be a multiple of the wavelength of light to satisfy the constructive interference condition. (Online version in colour.)

Because of the heterogeneity of plant tissue, it is also necessary to characterize the optical response of individual cells. Reflected and transmitted light from a microscopic scattering volume can be measured in a conoscopic configuration (figure 1*b*). Conoscopic imaging and spectroscopy are often performed using a microscope equipped with a Bertrand lens. In this configuration, it is possible to access the directionality of the scattered beam, i.e. to directly image the Fourier plane or the K-space distribution. Different directions (different colours in figure 1*b*) correspond to different points in the back focal plane (Fourier plane) of the lens L_1_. By using the system of lenses L_2_ and L_3_, it is possible to image the back focal plane of the first lens and thereby the Fourier components of the scattered light. Detection of the K-space image (P_4_ in figure 2*b*) by a camera images the scattered intensity distribution from the sample in Fourier space. In this technique, the lens L_1_ is generally an objective with a high numerical aperture (NA) in order to collect the largest number of scattered directions. In this configuration, it is important to control the NA of the light used to illuminate the sample. In the case of transmitted light, it is generally possible to vary the NA by varying the condenser. Depending on the type of structure analysed, it is convenient to use large or small NA: as an example if grating-like structures are considered, it is convenient to use light as collimated as possible, while for multilayer structures, where scattering is negligible, large NA provides directly specular reflection for different angles. In both the cases, the area of collection is defined by the field of view of the objective that is used. In reflection, the NA of illumination is defined by the objective L_1_; however, if the light is focused in the back focal plane P_2_ of the objective used, it is possible to also obtain collimated illumination by giving up part of the spatial resolution of the illumination.

The fibre core acts as a pinhole, selecting only a small area in K-space. By filtering the light collected from the fibre using a monochromator and a detector, it is possible to obtain spectra as a function of the position of the fibre, corresponding to different scattering directions. In order to obtain a large collection cone, it is necessary to use a high NA objective as lens L_1_.

A further device to record the complete hemispherical distribution of the scattered light is the imaging scatterometre [21]. In this set-up, the sample is placed in one of the two foci of an ellipsoidal mirror. The sample is illuminated through a small hole in the mirror. The backscattered light is reflected from the mirror and focused onto the second focus of the mirror into a narrow cone that is accepted by a photographic lens. The far-field scattering pattern in the back focal plane of this lens is imaged by a second lens onto a camera.

Another important factor to take into account for all the methods presented before is the light polarization of collection and illumination. In strongly scattering materials, the information of the polarization can be generally ignored: the multiple scattering process randomizes the polarization state of the incoming light. In the case of photonic structures, the polarization state of the reflected light provides interesting insight into the anatomy of the sample. Multilayer structures have different spectral response if illuminated with light with different linear polarization [22], whereas chiral multilayer structures selectively reflect light with specific handness [23].

### 2.2. Integrating sphere, backscattering and confocal spectroscopy

Whenever spatial information about the scattered light is not required, the overall optical response can be measured using an integrating sphere [24]. With this instrument, it is possible to measure the reflectivity/transmissivity of the sample integrated for all directions. The sphere consists of a hollow spherical cavity with a diffuse white reflective coating and several ports that are used to mount the sample, couple in the illumination beam and mount the detector. Light reflected from or transmitted through the sample is randomized by multiple scattering from the sphere walls and out-coupled using an optical fibre that is directly connected to a spectrometer. Similar light sources as for the goniometer experiment are used.

Backscattering spectroscopy is particularly advantageous for its flexibility. It employs fibre-probes consisting of six optical fibres that guide the light from the source to the sample. The six fibres are organized at the vertices of a hexagon, and they surround a further central fibre that collects the backscattered light and couples it into a spectrometer. While simple and flexible, this technique is limited in terms of spatial resolution.

Finally, in order to characterize the properties of photonic structures at the single cell level, it is necessary to use confocal microscopy, which allows the user to obtain spectral information from a single cell in an imaging mode [9]. Reflectance and transmittance measurements can be obtained both in bright-field and dark-field configurations using the halogen lamp of the microscope for illumination and an optical fibre mounted in the conjugate plane of the objective focal plane as a confocal pinhole. By varying the magnification and NA of the objective and the core of the fibre, a range of spatial resolutions can be obtained down to about 1 µm. This set-up also allows the insertion of additional optical elements into the beam path, such as polarizers or filters [11].

All spectroscopic methods have to be normalized against a reference. The characteristics of the samples and the spectral region in which the optical properties are studied define the choice of the appropriate reference. Commercially available white Lambertian reflectance standards (spectralon) are typically used to characterize strongly scattering samples in the UV-VIS and near-infrared region. On the other hand, aluminium and silver and gold mirrors are used to characterize samples with intense directional reflectivity, in the UV or VIS or near-infrared, respectively.

### 2.3. Anatomical and morphological characterization by electron microscopy

Because of the complex optical signature of plant tissues, it is typically necessary to investigate their anatomical morphology in order to understand the origin of the spectral response. Scanning electron microscopy (SEM) is therefore an indispensable tool, to start analysing the surface of the tissues, since it combines high spatial resolution with relatively simple sample preparation. The standard SEM preparation of biological samples requires chemical fixing to preserve and stabilize the structure of the tissue. A solution of glutaraldehyde is often used as fixative before dehydration by ethanol and critical point drying. To prevent charging by the electron beam, the samples are coated with noble metals, such as gold, using a sputter coater. However, fixing techniques sometimes damage the cuticle of the petal surface. Thus, it can often be advantageous to use cryo-SEM [25] in order to image fully hydrated biological tissues in their chemically unmodified state. Here, fresh samples attached to a stage are fixed by cooling using nitrogen slush before being sputter-coated with gold in the antechamber of the Cryo-SEM, maintaining the required low temperatures and avoiding the formation of ice crystals. Then, samples are introduced into the main chamber, where they can be imaged at a temperature below −100°C.

Alternatively, the surface morphology of a tissue can be examined by imaging a cast. One advantage of this method is that the tissue need not to be damaged, and indeed repeated casts can be made of the same tissue at different developmental stages or following different treatments. A two-step pattern transfer method is commonly used. A quick-setting viscous dental wax is cast onto the specimen to produce a negative replica. The dental wax replica is then used as a mould to create a positive epoxy replica of the specimen. The surface morphologies of these replicas are faithful down to the sub-100 nm length scale. Here too, SEM imaging requires a conductive surface coating of the replica.

SEM imaging allows the rapid examination of the surface of many samples, but its resolution can sometimes be insufficient to perform accurate measurement of photonic structures, especially when those are on the order of tens of nanometres. In addition, many plant tissues exhibit complex optical responses, and the presence of internal structures within the tissue (such as embedded nanocrystals or cell layers with specific properties) needs to be taken into account. Thus, the anatomy of biological tissues often needs to be further investigated using transmission electron microscopy (TEM) to image thin transverse sections of the plant tissue. Here, biological tissues are first chemically fixed and then embedded in a hard resin. Sections of a few micrometres in thickness are cut using an ultramicrotome before TEM examination. A range of methodologies is available in order to prepare samples for TEM analysis, but often the fixation, dehydratation and embedding processes can alter the organization and shape of the structures. Interestingly, cryofixation by high-pressure freezing could minimize such artefacts as this method vitrifies the biological samples to immobilize the molecular structures in their original state before imaging [26].

## 3. Photonic structures in flowers

Flowers are the reproductive structures of flowering plants (angiosperms) [27]. For pollination, many flowers attract animal pollinators by providing food rewards and using cues such as odour, temperature, colour or shape and size of different floral parts. The visual appearance of flowers is thus a crucial factor in interspecies communication that guarantees the reproductive success of a species. The petals are usually the most conspicuous parts of a flower, displaying vivid colour patterns that vary greatly between species. For this reason, studying how petal anatomy influences petal optical response is fundamental to understand the role of petals in evolution and to characterize the relationships between a given plant species and its pollinators [6,28–33]. In most angiosperms, flower coloration arises from pigments [34,35]. Changing the chemical nature of the pigments, varying their concentration and mixing them can all generate a broad colour palette. The intensity of the reflected colour, however, depends strongly on the shape of the epidermal cells containing the pigments [36]. Conical cells, for example, generally enhance colour brightness. The cell shape focuses the light onto the pigment-rich regions inside the cell, and it enhances scattering between neighbouring cells [13,30,36,37]. Structural colour in flowers arises mainly from surface diffraction gratings [6,8]. Diffraction gratings are periodical arrays of diffractive elements that periodically modulate the phase and/or amplitude of an incident light wave. In flowers, diffraction gratings consist of ordered striations or ridges that form on the epidermal cells. The formation of these striations during the development of the petals is not yet understood, but one possible mechanism is the buckling of the cuticle (a waxy layer that covers the surface of the plant epidermis) during anisotropic petal growth [38].

Diffraction gratings disperse monochromatic incident radiation into different angular directions, called orders. Light is thus not only specularly reflected (i.e. mirror-like, or zero-order diffraction, giving rise to gloss) but also additionally scattered in the plane perpendicular to the direction of the striations, according to the grating formula *a*(sin*θ*_*m*_ − sin*θ*_i_) = *m*λ**, where *θ*_i_ is the angle of incidence, *θ*_*m*_ is the angle of the *m*th scattered order, *λ* is the light wavelength and *a* is the grating periodicity.

The origin of the equation is illustrated in figure 2. For any wavelength *λ*, constructive interference requires the two scattered beams to be in phase, that is, their difference in travel path has to be a multiple of the wavelength: *m*λ**. From figure 2, this path difference is 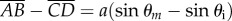.

While this equation applies for grating lines with transverse sections that are much smaller than *λ*, grating interference of less ideal gratings depends on the detailed shape of the grating line, requiring complex analytical or numerical approaches [39].

This simple equation however captures the essential picture. At any given value *θ*_i_, each wavelength *λ* is scattered into different angular directions. For incident white light, this gives rise to a rainbow-like colour dispersion in the direction perpendicular to the grating lines. The variation of this colour dispersion with the incident angle *θ*_i_ makes the grating surface iridescent.

**Figure 3.**
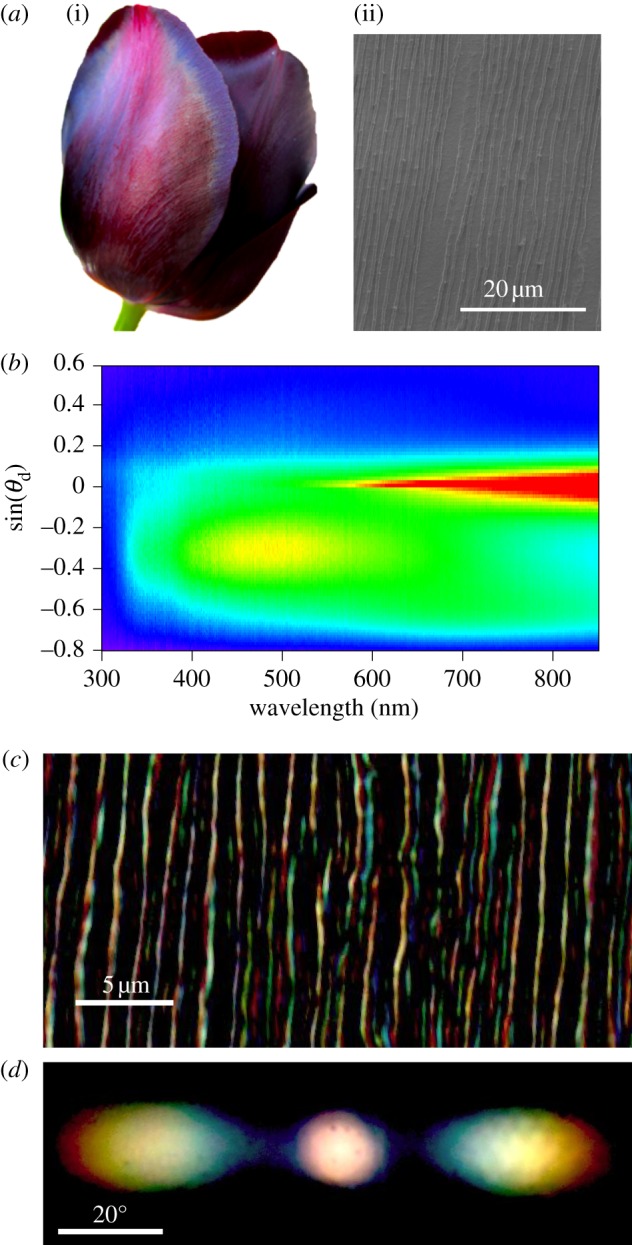
Striking iridescence of the tulip *Queen of the night*. (*a*) (i) Photograph of the flower adapted from [40]. The dark violet coloration is due to the pigment, whereas the blue appearance of the petal edge contains a contribution from the grating interference. (ii) Cryo-SEM image of the petal epidermis. The cells of the tulip epidermis are flat and uniaxially elongated; the cell dimension is approx. 80 × 20 µm^2^, while the distance between striation lines is ≈1 µm. (*b*) Optical spectrum of the epidermal layer obtained by the set-up as shown in figure 1*a* in reflection configuration for *θ*_i_ = 30°. The intensity is plotted on a violet-to-red colour-scale and the collection angle 0° corresponds to the specular reflection direction (sin(*θ*_d_) = 0). (*c*) Optical transmission microscopy image of the petal epidermis. (*d*) Diffraction pattern of the epidermis obtained with the set-up of figure 1*b* in transmission, adapted from [40].

The occurrence of natural diffraction gratings is surprisingly widespread in flowering plants. One of the most striking examples of iridescent coloration is observed in the tulip *Queen of the night* (figure 3*a*). The iridescent effect can be isolated from the underlying purple colour arising from the anthocyanin pigment by separating off the transparent epidermal layer. In many species of flowers, the epidermal layer is easy to peel off by using sharp tweezers and floating it onto a water surface. The epidermis is then transferred onto a planar substrate for optical characterization [9]. Figure 3*b,d* shows the optical signature of the epidermis of the tulip *Queen of the night* which is characteristic of a diffraction grating. Particularly in figure 3*b*, we note that the specular reflected signal is visible not only at 0°, but also at a range of angle between −10° and 10° indicating that the surface is not perfectly flat, while around −20°, (for sin(*θ*_d_) = 0.35) the first diffraction order is visible, also in this case the signal is really broad in angle due to disorder of the natural grating.

## 4. Photonic structures in leaves

The role of plant leaves is primarily photosynthesis, a process that allows plants to capture light and convert it into chemical energy that the organism can use [16,41]. The propagation of light inside leaves has been characterized theoretically and experimentally [37,42,43]. Depending on the illumination conditions in which the plant grows, leaves have different, and often quite sophisticated, mechanisms to optimize light capture or to protect themselves from intense UV radiation. For example, the curved epidermal cell walls on many tropical rainforest shade plants are thought to focus the light onto the photosynthetic layers within the leaf [13]. In another example, Edelweiss (*Leontopodium nivale*) uses photonic structures to protect the modified leaves (bracts) that surround its reproductive organs. In high altitudes where high UV exposure may be harmful to plant tissues, Edelweiss has developed a filamentous woolly layer composed of nano-structured fibres on its bracts, which reduces the UV intensity reaching internal tissues by multiple scattering [44]. Alternatively, thin wax layers on the needles of blue spruce trees (*Picea pungens*) [45] and on the leaves of *Dudleya brittonii* [15] are particularly efficient in UV screening.

More complex structures can be found in the epidermal layers of leaves of *Selaginella willdenowii* and *Begonia pavonina*, producing iridescence in the UV and blue. The biological function of this iridescence, if any, is not well understood yet [46,47]. The underlying mechanism arises either from multilayer interference of two or more materials with different refractive indexes [10,48,49], or by stacking layers of cellulose microfibrils with differing orientations forming a helicoid structure similar to a liquid crystal nematic phase [46,50–52].

**Figure 4.**
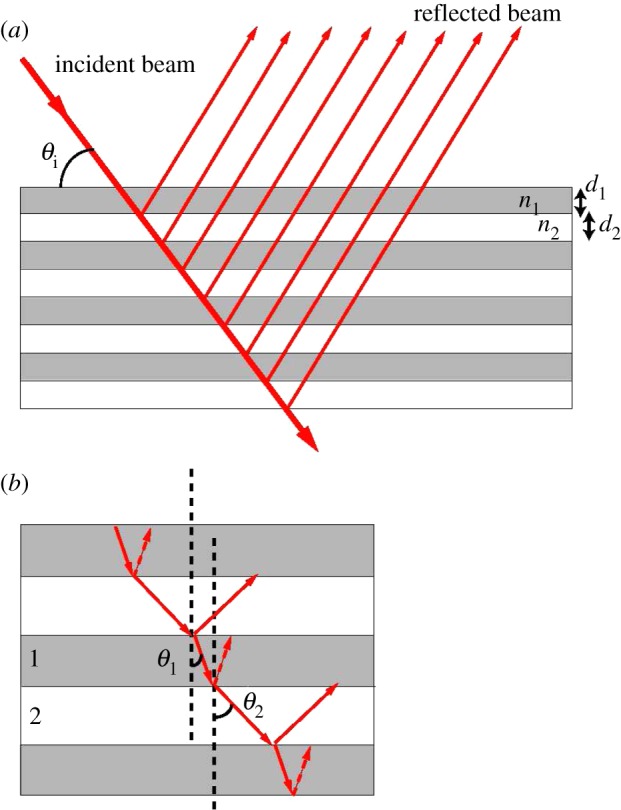
Multilayer interference mechanism. (*a*) Incident beam is reflected at the interface between layers of different materials, represented with different colours. (*b*) Zoomed image of the light reflection–refraction at the interface between layers. (Online version in colour.)

The principle of multilayer interference of light is illustrated in figure 4. Incident light is reflected at each interface between two materials of different refractive indices. Depending on the wavelength and on the angle of incidence, the reflected beams interfere constructively or destructively. The multilayer therefore acts as a colour filter, reflecting a certain colour (wavelength range) while transmitting the complementary colour. Consider a multilayer made of two materials with thicknesses *d*_1_ and *d*_2_ and refractive indices *n*_1_ and *n*_2_ (*n*_1_ > *n*_2_). Maximal constructive interference occurs when two conditions are simultaneously fulfilled: (i) the phase difference of a beam traversing a double layer of both materials (compared with a freely propagating beam) is a multiple of the wavelength. This corresponds to the condition 1(*n*_1_*d*_1_cos*θ*_1_ + *n*_2_*d*_2_cos*θ*_2_) = *m*λ** and (ii) phase difference of two beams reflected at the interface between material 1 and 2 satisfies the relation 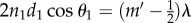, with the additional condition of 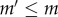 [22]. Selective wavelength reflection can however be achieved with multilayers that do not fully satisfy both conditions, i.e. for different thicknesses and refractive indices. The optical response of arbitrary multilayer stacks is generally calculated using the transfer matrix approach [53].

**Figure 5.**
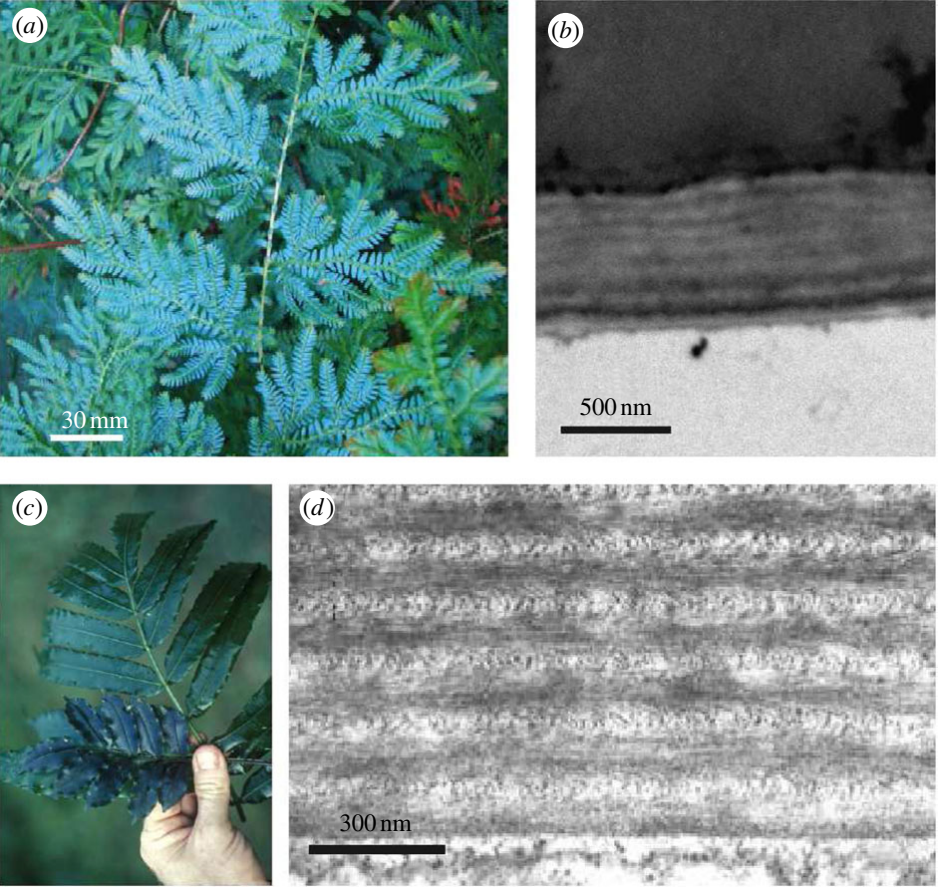
(*a*) Photograph of a juvenile *Selaginella willdenowii* leave. (*b*) TEM transverse section image of the outer cell wall and cuticle of the upper epidermis of a juvenile blue leaf. (*a,b*) Reproduced with permission, 2010 The Royal Society [10]. (*c*) Photograph of a juvenile (blue) and adult (green) leaf of *Danaea nodosa*. (*d*) TEM transverse section image of the outer cell wall of a juvenile leave. (*c,d*) Reproduced with permission from [16].

In plants, multilayers can be found either at the surface of the leaves (on top of the epidermis) or within specialized intracellular organelles, the so-called iridoplasts, which are located inside the cells of the upper epidermis. One example is the young leaves of *Selaginella willdenowii* [10,54,55] in figure 5*a*, displaying brilliant blue coloration. TEM transverse section imaging of a blue leaf reveals a layered structure at the outer edge of the cell wall of the upper epidermis (figure 5*b*). By contrast, the blue iridescence in *Begonia pavonina*, a rainforest understory plant, arises from the iridoplasts located inside the epidermal cells [46].

**Figure 6.**
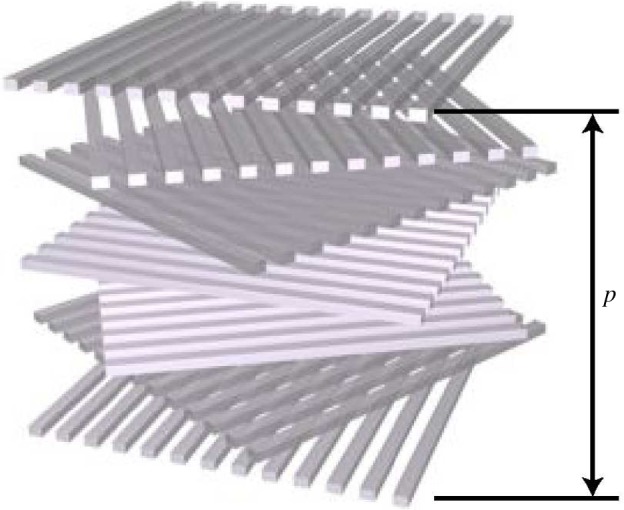
Schematic of a helicoidal stack. Cellulose microfibrils are oriented parallel to each other forming a plane. The planes are superposed with a small rotation angle. The distance *p* between layers of fibrils with the same orientation determines the reflected wavelength, while the rotation direction determines the circular polarization of the reflected light. (Online version in colour.)

A second commonly observed mechanism to produce UV–blue light iridescence makes use of only one single material, cellulose microfibrils, which are spatially organized to form a helicoid architecture, schematically shown in figure 6. Sheets of cellulose fibrils deposited parallel to each other are inherently birefringent due to the asymmetry of the system. Since the orientation of the microfibrils in successive layers is rotated by a constant angle, both linear components of a light wave experience a change of refractive index when passing from one layer to the next, generating the reflection of circular polarized light with opposite helicoidicity to the rotating stack. The distance *p* over which the fibrils in the planes have the same orientation defines the periodicity of the multilayer, and therefore the range of wavelengths *λ* that are reflected by the stack. In the simplified case where the difference between the refractive index of the cellulose fibrils and the matrix in which they are embedded is low, the maximum reflectivity is obtained for *λ* = 2*np*, where *n* is the refractive index of the fibrils [23].

Figure 5*c* shows the blue iridescence of *Danaea nodosa*. The transverse section TEM image of a juvenile blue leaf in figure 5*d* reveals the helicoidal structure in the cell walls of the epidermis. For a value of *p* of about 160 nm and taking *n* = 1.53 of dried cellulose [56], a blue–green reflection is obtained with the above equation [50].

## 5. Photonic structures in fruits

**Figure 7.**
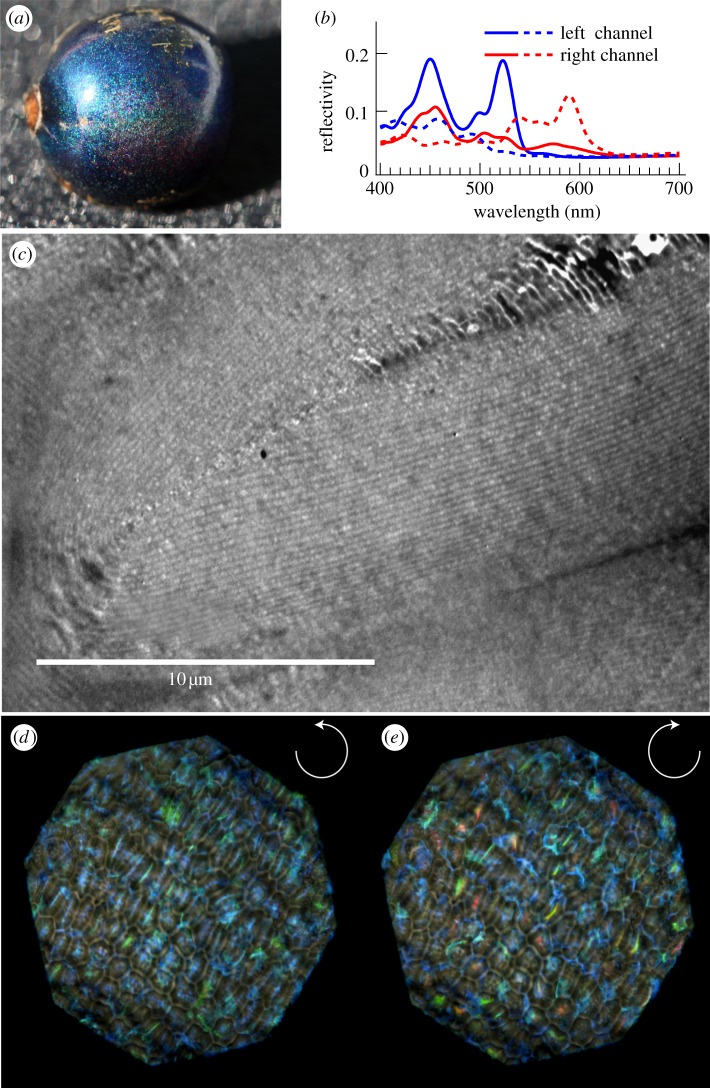
(*a*) Photograph of a *Pollia condensata* fruit. The colour of the fruit arises from the interference of light and not from pigment. (*b*) Spectra from two different cells (continuous and dotted lines, respectively) for the two polarization channels (red and blue colour, respectively). The double-peak structure arises from the helicoidal stacks in the epicarp cells. The varying peak positions are indicative of varying values of the stack pitch *p*. (*c*) Transverse section TEM image of the multilayered cell wall that gives rise to the blue colour. (*d,e*) Optical microscopy images of the fruit in epi-illumination in circularly left and right polarization channels, respectively. (Reproduced with permission from [11].)

**Figure 8.**
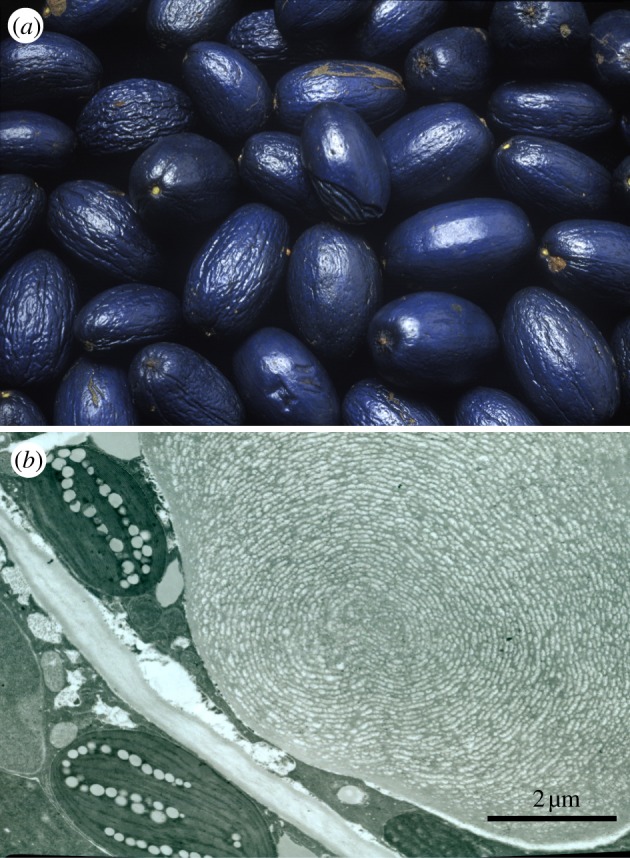
(*a*) Photograph of *Delarbrea michieana* fruits. The whole fruit is about 1.7 cm long. (*b*) TEM transverse section image of the fruit, where an iridosome responsible for the fruit coloration is visible at the right-hand side of the image. (Reproduced with permission from [16].)

Helicoidal cellulose-based stacks are also found in fruits. Plants aim to attract seed dispersers by providing a nutritious reward, such as the fruit pulp. Some plants, however, deceive their seed dispersers by producing fruits that visually mimic the appearance of fleshy pulp-rich fruits of other species growing nearby but do not offer any nutritional reward [57]. The fruits of such plants can display a striking brilliant coloration that is used as an advertisement to attract animals [58]. The fruits of *Pollia condensata* (figure 7*a*) for instance, constitute an interesting example [11]. The blue colour of the fruit arises from a cellulose-based helicoidal stack similar to the one found in the leaves of *Danaea nodosa*. In *Pollia*, the cellulose stack is found in the epidermal cell wall. A TEM transverse section image of the *Pollia* fruit reveals that the cellulose helicoidal structure occupies most of the cell wall that surrounds each cell of the epicarp (figure 7*c*). Remarkably, the stack structure and therefore the strong coloration remain intact in the dry fruit. The fruit in figure 7*a* is more than 50 years old and has the same appearance as fresh ones. When examining the fruit in epi-illumination using circular polarization filters, the variation in coloration of each cell is revealed, corresponding to slightly varying values of the pitch *p* in the different cells. It is important to note that each cell shows colour in a specific polarization channel (figure 7*d,e*), meaning that each cell has a specific handness of the helicoidal structures in the cell wall. The average reflectivity is about 30% with respect to a silver mirror, which is almost one order of magnitude larger than the reflectivity that can be obtained by pigmentation. Similar stack structures have been found in fruits of *Margaritaria nobilis*, showing strong iridescence in the blue–green region of the spectrum [12,17] that gives the fruit a strong metallic appearance. In this case, the use of an optical microscope equipped with circularly polarized filters is particularly useful in order to understand the anatomy of the structure under consideration. The fact that each cell reflects selectively left- or right-handed circularly polarized light provides really good insight into the characteristics of the photonic structure. A further example of multilayer interference is found in the fruit of *Elaeocarpus angustifolius*. In this case, the coloration of the fruit arises from the presence of iridosomes in mature fruits. These iridosomes consist of polysaccharide layers (including cellulose) probably secreted by the cytoplasm of the epidermal cell, forming a three-dimensional lattice underneath the outer cell wall of the upper epidermis, in direct contact with the cell membrane [59]. In the case of the blue coloration of *Delarbrea michieana* fruits the colour is produced by similar iridosomes [48] (figure 8).

## 6. Conclusion

Plant anatomy is directly influenced by the light conditions in which plants grow. Plants exploit light to harvest solar energy through photosynthesis and to communicate with animals. Consequently, the characterization of their optical properties is important in understanding the evolution of the interplay between plants and the animals that pollinate them or disperse their seeds. Structural colour and the photonic structures that produce it must be characterized in order to describe fully the optical response of different species of plants. This review summarizes and discusses a range of mechanisms used by plants to produce structural colour and the experimental methods now available to study them. In contrast to animals, structural colour in plants has been little investigated. This provides scope for the discovery of additional photonic mechanisms, given the enormous morphological diversity of the more than 300 000 angiosperm species. This review also highlights the paucity of knowledge of the possible biological functions of many of these photonic structures. Is the production of structural colour the primary function of a periodic morphology or is the visual effect simply a by-product of a structure that fulfils a different, non-optical function? Finally, almost nothing is known about the developmental mechanisms and the genetic controls used by organisms, plants or animals, to produce these precisely organized microstructures. The interaction of scientists from a range of disciplines is required to fully understand the significance of this fascinating optical phenomenon.
